# 2-(Naphthalen-2-yl­oxy)-*N*′-[2-(naphthalen-2-yl­oxy)acet­yl]acetohydrazide monohydrate

**DOI:** 10.1107/S241431462100314X

**Published:** 2021-03-31

**Authors:** Gamal A El-Hiti, Bakr F. Abdel-Wahab, Mohammed A. Baashen, Emad Yousif, Amany S. Hegazy, Benson M. Kariuki

**Affiliations:** a Cornea Research Chair, Department of Optometry, College of Applied, Medical Sciences, King Saud University, PO Box 10219, Riyadh 11433, Saudi Arabia; b Applied Organic Chemistry Department, National Research Centre, Dokki, Giza 12622, Egypt; c Department of Chemistry, College of Science and Humanities, Shaqra University, Al-Dawadmi 11911, Saudi Arabia; d Department of Chemistry, College of Science, Al-Nahrain University, Baghdad, 64021, Iraq; e School of Chemistry, Cardiff University, Main Building, Park Place, Cardiff CF10 3AT, UK; Goethe-Universität Frankfurt, Germany

**Keywords:** crystal structure, hydrazide, naphthalene, acyl­hydrazines

## Abstract

The title compound, C_24_H_20_N_2_O_4_·H_2_O, crystallizes with half a mol­ecule of 2-(naphthalen-2-yl­oxy)-*N*′-[2-(naphthalen-2-yl­oxy)acet­yl]acetohydrazide and half a water mol­ecule in the asymmetric unit. In the crystal, mol­ecules form planes parallel to (011). Two mol­ecules are connected by water mol­ecules *via* O—H⋯O and N—H⋯O hydrogen bonds.

## Abstract 2

In the crystal, mol­ecules of the title compound, C_24_H_20_N_2_O_4_·H_2_O, form twisted planes parallel to (011). Water mol­ecules donate O—H⋯O and accept N—H⋯O hydrogen bonds, forming helical arrangements.

## Graphical abstract

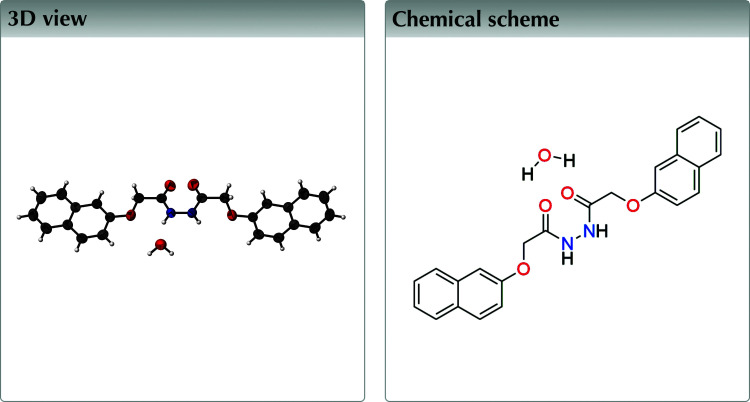

## Structure description

Di­acyl­hydrazines can be used as environmentally friendly insecticides against lepidopteran larvae and ground-dwelling coleopterans (Morou *et al.*, 2013 ▸; Suzuki *et al.*, 2017 ▸; Wang *et al.*, 2017 ▸). In addition, they are precursors in the synthesis of electroluminescent devices (Huang *et al.*, 2009 ▸; Wu & Chen, 2009 ▸, 2010 ▸).

**Figure 1:**
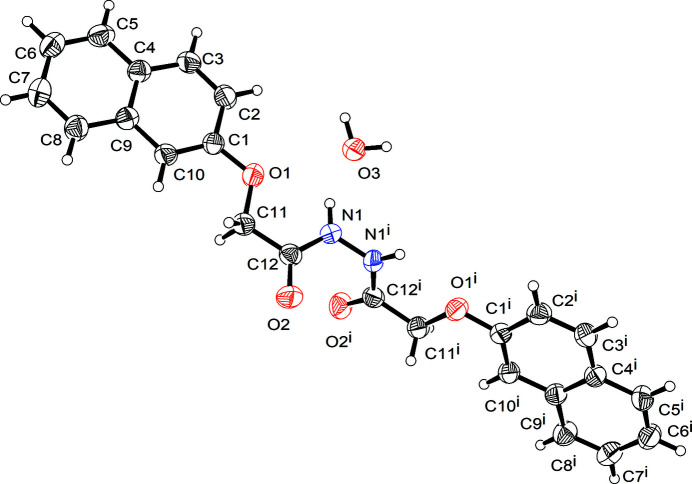
The mol­ecular structure of the title compound showing 50% displacement ellipsoids.

The asymmetric unit comprises half a mol­ecule of 2-(naphthalen-2-yl­oxy)-*N*′-[2-(naphthalen-2-yl­oxy)acet­yl]acetohydrazide and half a mol­ecule of water, both centred on the twofold rotation axis parallel to the *c* axis. An *ORTEP* representation is shown in Fig. 1 ▸. Similar to 2-[(naphthalen-2-yl)­oxy]acetamide (Huang *et al.*, 2020 ▸), the 2-[(naphthalen-2-yl)­oxy]acetamidyl unit of the title compound is planar, and the twist angle between the two halves is 64.9 (1)°.

**Figure 2:**
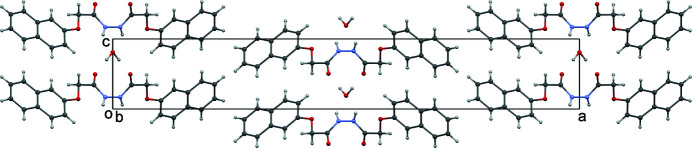
The crystal structure viewed down the *b* axis.

**Figure 3:**
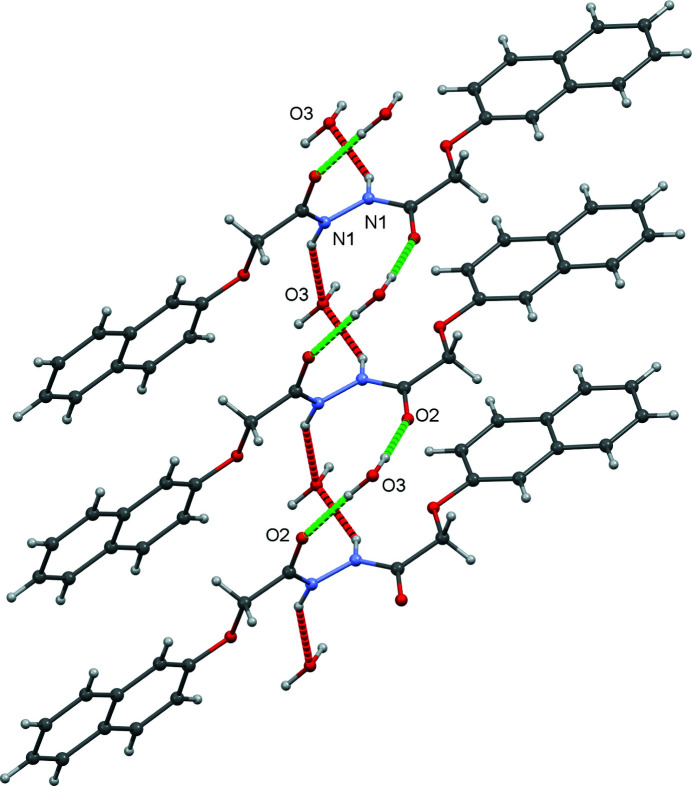
A segment of the crystal structure showing O—H⋯O hydrogen bonds as green dashed lines and N—H⋯O inter­actions as red dashed lines.

In the crystal, the mol­ecules form planes parallel to (011) (Fig. 2 ▸). Two 2-(naphthalen-2-yl­oxy)-*N*′-[2-(naphthalen-2-yl­oxy)acet­yl]acetohydrazide mol­ecules are connected by a water mol­ecule *via* O—H⋯O and N—H⋯O bonds (Fig. 3 ▸, Table 1 ▸). One water mol­ecule donates O—H⋯O hydrogen bonds to two neighbouring mol­ecules (related by twofold rotation), leading to the formation of a helix parallel to the *c* axis (green dashed lines in Fig. 3 ▸). The same pair of mol­ecules is also connected by N—H⋯O bonds, resulting in a second parallel helical arrangement (red dashed lines in Fig. 3 ▸).

## Synthesis and crystallization

A mixture of ethyl 2-cyano-3-eth­oxy­acrylate (0.34 g, 2.0 mmol) and 2-(naphthalen-2-yl­oxy)acetohydrazide (0.43 g, 2.0 mmol) in dry ethanol (10 mL) was heated with stirring under reflux for 2 h. The solid formed on cooling to room temperature. It was collected by filtration, washed with ethanol, dried and recrystallized from di­methyl­formamide to give colourless crystals, m.p. > 300°C (lit. m.p. > 300°C; Abdel-Wahab *et al.*, 2017 ▸) of the title compound in 76% yield.

## Refinement

Crystal data, data collection and structure refinement details are summarized in Table 2 ▸.

## Supplementary Material

Crystal structure: contains datablock(s) I. DOI: 10.1107/S241431462100314X/bt4110sup1.cif

Structure factors: contains datablock(s) I. DOI: 10.1107/S241431462100314X/bt4110Isup2.hkl

Supporting information file. DOI: 10.1107/S241431462100314X/bt4110Isup3.cml

CCDC reference: 2072979

Additional supporting information:  crystallographic information; 3D view; checkCIF report

## Figures and Tables

**Table 1:** Hydrogen-bond geometry (Å, °)

*D*—H⋯*A*	*D*—H	H⋯*A*	*D*⋯*A*	*D*—H⋯*A*
N1—H1⋯O3	0.86	2.19	2.954 (6)	149
O3—H1*O*⋯O2^i^	0.92 (6)	1.87 (6)	2.796 (5)	177 (6)

Symmetry code: (i) 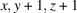 .

**Table 2:** Experimental details

Crystal data	
Chemical formula	C_24_H_20_N_2_O_4_·H_2_O
*M*_r_	418.43
Crystal system, space group	Orthorhombic, *P*2_1_2_1_2
Temperature (K)	293
*a*, *b*, *c* (Å)	37.196 (4), 4.8441 (4), 5.5840 (4)
*V* (Å^3^)	1006.12 (15)
*Z*	2
Radiation type	Cu *K*α
μ (mm^−1^)	0.80
Crystal size (mm)	0.37 × 0.05 × 0.02

Data collection	
Diffractometer	Rigaku Oxford Diffraction SuperNova, Dual, Cu at home/near, Atlas
Absorption correction	Gaussian (*CrysAlis PRO*; Rigaku OD, 2015 ▸)
*T*_min_, *T*_max_	0.687, 1.000
No. of measured, independent and observed [*I* > 2σ(*I*)] reflections	7482, 2079, 1456
*R*_int_	0.063
(sin θ/λ)_max_ (Å^−1^)	0.632

Refinement	
*R*[*F* ^2^ > 2σ(*F* ^2^)], *wR*(*F* ^2^), *S*	0.062, 0.172, 1.05
No. of reflections	2079
No. of parameters	145
H-atom treatment	H atoms treated by a mixture of independent and constrained refinement
Δρ_max_, Δρ_min_ (e Å^−3^)	0.31, −0.19
Absolute structure	Flack *x* determined using 424 quotients [(*I* ^+^)−(*I* ^−^)]/[(*I* ^+^)+(*I* ^−^)] (Parsons *et al.*, 2013 ▸)
Absolute structure parameter	0.1 (3)

Computer programs: *CrysAlis PRO* (Rigaku OD, 2015 ▸), *SHELXS* (Sheldrick, 2008 ▸), *SHELXL2018* (Sheldrick, 2015 ▸), *ORTEP-3 for Windows* (Farrugia, 2012 ▸), *Mercury* (Macrae *et al.*, 2020 ▸) and *CHEMDRAW Ultra* (Cambridge Soft, 2016 ▸).

## full crystallographic data

### Crystal data

**Table:**

C_24_H_20_N_2_O_4_·H_2_O	*D*_x_ = 1.381 Mg m^−^^3^
*M**_r_* = 418.43	Cu *K*α radiation, λ = 1.54184 Å
Orthorhombic, *P*2_1_2_1_2	Cell parameters from 1274 reflections
*a* = 37.196 (4) Å	θ = 4.7–73.7°
*b* = 4.8441 (4) Å	µ = 0.80 mm^−^^1^
*c* = 5.5840 (4) Å	*T* = 293 K
*V* = 1006.12 (15) Å^3^	Needle, colourless
*Z* = 2	0.37 × 0.05 × 0.02 mm
*F*(000) = 440	

### Data collection

**Table:**

Rigaku Oxford Diffraction SuperNova, Dual, Cu at home/near, Atlas diffractometer	1456 reflections with *I* > 2σ(*I*)
ω scans	*R*_int_ = 0.063
Absorption correction: gaussian (CrysAlisPro; Rigaku OD, 2015)	θ_max_ = 76.9°, θ_min_ = 4.8°
*T*_min_ = 0.687, *T*_max_ = 1.000	*h* = −47→44
7482 measured reflections	*k* = −6→5
2079 independent reflections	*l* = −4→6

### Refinement

**Table:**

Refinement on *F*^2^	Hydrogen site location: mixed
Least-squares matrix: full	H atoms treated by a mixture of independent and constrained refinement
*R*[*F*^2^ > 2σ(*F*^2^)] = 0.062	*w* = 1/[σ^2^(*F*_o_^2^) + (0.0593*P*)^2^ + 0.4016*P*] where *P* = (*F*_o_^2^ + 2*F*_c_^2^)/3
*wR*(*F*^2^) = 0.172	(Δ/σ)_max_ < 0.001
*S* = 1.05	Δρ_max_ = 0.31 e Å^−^^3^
2079 reflections	Δρ_min_ = −0.19 e Å^−^^3^
145 parameters	Absolute structure: Flack *x* determined using 424 quotients [(*I*^+^)-(*I*^-^)]/[(*I*^+^)+(*I*^-^)] (Parsons *et al.*, 2013)
0 restraints	Absolute structure parameter: 0.1 (3)

### Special details

**Table:**

Geometry. All esds (except the esd in the dihedral angle between two l.s. planes) are estimated using the full covariance matrix. The cell esds are taken into account individually in the estimation of esds in distances, angles and torsion angles; correlations between esds in cell parameters are only used when they are defined by crystal symmetry. An approximate (isotropic) treatment of cell esds is used for estimating esds involving l.s. planes.
Refinement. The coordinates for the water hydrogen atom was refined freely. The rest of the hydrogen atoms were positioned geometrically and refined using a riding model. All *U*_iso_(H) were constrained to be 1.2 times *U*_eq_(C,*N*).

### Fractional atomic coordinates and isotropic or equivalent isotropic displacement parameters (Å^2^)

**Table:**

	*x*	*y*	*z*	*U*_iso_*/*U*_eq_	
C1	0.39660 (13)	0.5528 (10)	0.8789 (8)	0.0464 (10)	
C2	0.39748 (15)	0.7245 (12)	1.0831 (9)	0.0555 (12)	
H2	0.416807	0.717218	1.188528	0.067*	
C3	0.36943 (15)	0.9021 (12)	1.1238 (9)	0.0571 (13)	
H3	0.370140	1.016895	1.257183	0.068*	
C4	0.33940 (14)	0.9153 (10)	0.9681 (9)	0.0501 (11)	
C5	0.31024 (16)	1.0968 (12)	1.0072 (11)	0.0616 (14)	
H5	0.310336	1.210702	1.141216	0.074*	
C6	0.28194 (15)	1.1080 (12)	0.8520 (11)	0.0635 (14)	
H6	0.262865	1.227610	0.880591	0.076*	
C7	0.28187 (15)	0.9368 (12)	0.6482 (12)	0.0642 (14)	
H7	0.262674	0.944144	0.541710	0.077*	
C8	0.30967 (15)	0.7608 (12)	0.6059 (10)	0.0595 (13)	
H8	0.309150	0.649189	0.470417	0.071*	
C9	0.33923 (14)	0.7443 (11)	0.7632 (9)	0.0480 (11)	
C10	0.36861 (14)	0.5619 (11)	0.7222 (9)	0.0488 (11)	
H10	0.368669	0.448574	0.587834	0.059*	
C11	0.42712 (13)	0.2082 (10)	0.6538 (9)	0.0499 (11)	
H11A	0.427237	0.319235	0.509307	0.060*	
H11B	0.405620	0.094828	0.651848	0.060*	
C12	0.45969 (14)	0.0248 (10)	0.6558 (8)	0.0477 (10)	
O2	0.46226 (11)	−0.1567 (8)	0.5050 (8)	0.0639 (10)	
O3	0.500000	0.500000	1.1908 (10)	0.0627 (14)	
O1	0.42622 (10)	0.3845 (8)	0.8567 (7)	0.0549 (9)	
N1	0.48388 (11)	0.0727 (9)	0.8289 (7)	0.0494 (9)	
H1	0.479501	0.192285	0.938948	0.059*	
H1O	0.4869 (17)	0.609 (14)	1.295 (11)	0.069 (19)*	

### Atomic displacement parameters (Å^2^)

**Table:**

	*U*^11^	*U*^22^	*U*^33^	*U*^12^	*U*^13^	*U*^23^
C1	0.051 (3)	0.044 (2)	0.045 (2)	0.004 (2)	0.0000 (19)	0.0022 (18)
C2	0.061 (3)	0.055 (3)	0.050 (3)	0.003 (2)	−0.009 (2)	0.000 (2)
C3	0.068 (3)	0.056 (3)	0.047 (3)	0.008 (2)	−0.004 (2)	−0.011 (2)
C4	0.057 (3)	0.043 (2)	0.050 (3)	0.004 (2)	0.004 (2)	0.002 (2)
C5	0.074 (3)	0.053 (3)	0.058 (3)	0.010 (3)	0.011 (3)	−0.003 (3)
C6	0.057 (3)	0.058 (3)	0.075 (4)	0.013 (3)	0.007 (3)	0.004 (3)
C7	0.055 (3)	0.060 (3)	0.078 (4)	0.007 (3)	−0.006 (3)	0.008 (3)
C8	0.056 (3)	0.057 (3)	0.066 (3)	0.003 (2)	−0.009 (2)	−0.006 (2)
C9	0.052 (3)	0.042 (2)	0.050 (2)	0.001 (2)	0.0030 (19)	0.0036 (19)
C10	0.056 (3)	0.044 (2)	0.046 (2)	0.004 (2)	−0.0023 (19)	−0.0042 (18)
C11	0.055 (3)	0.047 (2)	0.048 (3)	0.003 (2)	−0.003 (2)	−0.003 (2)
C12	0.055 (3)	0.045 (2)	0.042 (2)	0.001 (2)	0.001 (2)	0.0014 (19)
O2	0.068 (2)	0.060 (2)	0.063 (2)	0.0081 (19)	−0.003 (2)	−0.0190 (19)
O3	0.075 (4)	0.060 (3)	0.053 (3)	0.015 (3)	0.000	0.000
O1	0.0528 (19)	0.0537 (19)	0.058 (2)	0.0103 (15)	−0.0078 (16)	−0.0032 (17)
N1	0.050 (2)	0.054 (2)	0.045 (2)	0.0109 (18)	0.0001 (18)	−0.0065 (18)

### Geometric parameters (Å, º)

**Table:**

C1—C10	1.361 (7)	C7—H7	0.9300
C1—O1	1.376 (6)	C8—C9	1.410 (7)
C1—C2	1.412 (7)	C8—H8	0.9300
C2—C3	1.371 (8)	C9—C10	1.424 (7)
C2—H2	0.9300	C10—H10	0.9300
C3—C4	1.417 (7)	C11—O1	1.419 (6)
C3—H3	0.9300	C11—C12	1.502 (7)
C4—C9	1.413 (7)	C11—H11A	0.9700
C4—C5	1.413 (7)	C11—H11B	0.9700
C5—C6	1.364 (9)	C12—O2	1.221 (6)
C5—H5	0.9300	C12—N1	1.341 (6)
C6—C7	1.408 (9)	O3—H1O	0.92 (6)
C6—H6	0.9300	N1—N1^i^	1.391 (8)
C7—C8	1.361 (8)	N1—H1	0.8600
			
C10—C1—O1	125.0 (4)	C7—C8—H8	119.3
C10—C1—C2	121.2 (5)	C9—C8—H8	119.3
O1—C1—C2	113.8 (4)	C8—C9—C4	118.4 (5)
C3—C2—C1	119.1 (5)	C8—C9—C10	122.3 (5)
C3—C2—H2	120.5	C4—C9—C10	119.4 (5)
C1—C2—H2	120.5	C1—C10—C9	120.3 (5)
C2—C3—C4	121.8 (5)	C1—C10—H10	119.9
C2—C3—H3	119.1	C9—C10—H10	119.9
C4—C3—H3	119.1	O1—C11—C12	111.7 (4)
C9—C4—C5	119.1 (5)	O1—C11—H11A	109.3
C9—C4—C3	118.3 (5)	C12—C11—H11A	109.3
C5—C4—C3	122.6 (5)	O1—C11—H11B	109.3
C6—C5—C4	121.3 (5)	C12—C11—H11B	109.3
C6—C5—H5	119.4	H11A—C11—H11B	107.9
C4—C5—H5	119.4	O2—C12—N1	124.7 (5)
C5—C6—C7	119.4 (5)	O2—C12—C11	118.9 (5)
C5—C6—H6	120.3	N1—C12—C11	116.4 (4)
C7—C6—H6	120.3	C1—O1—C11	116.6 (4)
C8—C7—C6	120.5 (5)	C12—N1—N1^i^	119.4 (4)
C8—C7—H7	119.7	C12—N1—H1	120.3
C6—C7—H7	119.7	N1^i^—N1—H1	120.3
C7—C8—C9	121.3 (5)		

Symmetry code: (i) −*x*+1, −*y*, *z*.

### Hydrogen-bond geometry (Å, º)

**Table:**

*D*—H···*A*	*D*—H	H···*A*	*D*···*A*	*D*—H···*A*
N1—H1···O3	0.86	2.19	2.954 (6)	149
O3—H1*O*···O2^ii^	0.92 (6)	1.87 (6)	2.796 (5)	177 (6)

Symmetry code: (ii) *x*, *y*+1, *z*+1.
